# Action Research on Applying Compound Stimulus Approach to Improve Empathetic Communication: The Case of Physical Therapy Students

**DOI:** 10.3390/healthcare11040553

**Published:** 2023-02-13

**Authors:** Yu-Hsiu Chu, Shin-Yi Lee, Yao-Chuen Li, Shu-Ya Chen, Wei-Fen Ma

**Affiliations:** 1 Department of Physical Therapy and Graduate Institute of Rehabilitation Science, China Medical University, Taichung 406040, Taiwan; 2 Foreign Language Center, Feng Chia University, Taichung 407102, Taiwan; 3 School of Nursing, China Medical University, Taichung 406040, Taiwan; 4 Nursing Department, China Medical University Hospital, Taichung 404332, Taiwan; 5 PhD Program in Healthcare Science, College of Health Care, China Medical University, Taichung 406404, Taiwan

**Keywords:** empathy, communication, physical therapy, drama in education, compound stimulus

## Abstract

(1) Background: Empathetic communicative skills are the first step in establishing a good therapeutic relationship. The purpose of this study is to understand the effectiveness of improving the empathetic communicative skills applied to obtain accurate and precise information from patients via compound stimulus-drama in education. (2) Methods: A cross-sectional, one-group, pre- and post-test design was used for this study. In the two-day workshop, four clinical physiotherapists acted as tutors for the “Compound Stimulus-Drama in Education” module and assessed students’ performances. The Standard Patient Rating Scale (SPRS), Objective Structured Clinical Examination Scale (OSCES), Professional and Communication Self-Assessment Scale (PCSS), Patients’ Information (PI), and the Jefferson Scale of Empathy (JSE) were used to assess the students’ empathy scores and communication skills, before and after the course. (3) Results: Fifty-seven students participated in this study. The results showed that there were significant improvements in the SPRS, OSCES, PCSS, PI, and JSE (*p* < 0.05). Both the quantitative data and the participants’ reflection feedback suggest that this novel module was more helpful than traditional clinical practice courses for improving clinical empathy communication skills. (4) Conclusions: This study provided an innovative teaching model and assessment tools for learning clinic empathetic communicative skills in future education training.

## 1. Introduction

Along with the rise in the demand of the general public for health improvement, disease prevention, and long-term care, physical therapists have come to play an increasingly significant role in healthcare systems worldwide [1]. In 2022, the American Physical Therapy Association (APTA) proposed the following seven core values for physical therapists: accountability, altruism, compassion/caring, excellence, integrity, professional duty, and social responsibility [2]. Compassionate and caring physical therapists show and practice “concern, empathy, and consideration for the needs and values of others” (APTA, 2022). World Physiotherapy (WP) considers “communication” a core competence of physiotherapists, accentuating the need to “demonstrate reflecting listening and negotiating skills to develop trust and to enhance relationships and outcomes with patients, clients, and other colleagues” [3]. In Taiwan, communication skills, alongside professional expertise, were recognized as an essential competence of PT graduates as early as in the Physical Therapy Education Symposium held in 1997 [2]. Therefore, it is obvious that physical therapists currently need to demonstrate adequate communication skills and empathy. Based on their real-life experiences, clinical PT practitioners have been recommending PT graduates to equip themselves with self-reflection and critical thinking skills that are essential for developing the “soft power” of communication and coordination [2,4]. Accordingly, greater emphasis has been placed on incorporating the cultivation of students’ empathetic, caring, and communication capacities as an integral part of the goals of PT education [5,6]. However, while it is heartening to find that most PT departments and graduate institutes in Taiwan respond to the need of developing students’ communication skills, training for building empathy has not been receiving the attention it deserves. As a result, this study aimed to adopt an innovative teaching approach to help PT students improve their communication skills and develop their empathetic capacity, learning how to obtain precise information with efficiency to achieve ongoing enhancement of their professional competence.

The word “communication” derives from the Latin word commūnicātiōnem (literally meaning “to make common”) and means, by extension, “to exchange data or information between entities” in order to resolve conflicts and reach agreement [7]. “Empathy,” on the other hand, is the capacity to understand or feel what another person is experiencing; the willingness or ability to step out of self-centered parochialism to see and examine things from another person’s perspective; and to imagine what it would be like to be in that person’s position [8]. Together, “empathetic communication” means understanding and accepting another person’s feelings first, and then proceeding to initiate interaction and build trust through effective communication [9]. For medical professionals, empathetic communication requires them to eschew making arbitrary judgments on patients based on individual preferences or convenience. Instead, objective observation is necessary to better identify and respond to patients’ needs, and to treat patients with a sincere, caring, positive, and optimistic attitude characterized by equality and mutual respect [9].

According to previous studies, good communication skills form a major factor affecting patient satisfaction, patient–provider relationships, and clinical outcomes [10,11,12]. Empathy is also a key factor of therapeutic efficacy; a physical therapist capable of demonstrating empathy through perspective taking is more likely to establish therapeutic alliances with patients [13].

However, while studies that traced empathy among medical students have not been able to identify a conclusive relationship between empathy scores and educational level [14,15], medical students with a higher empathy score in the preclinical years are more likely to demonstrate empathy in the clinical years [16]. Strengthening empathy training in the preclinical years can, therefore, be expected to improve clinical empathy. This study adopted an innovative teaching approach based on the core concepts of compound stimulus [17] to enhance the empathetic communication skills of PT students through interactive theater.

The practice of PT requires direct, face-to-face interactions with patients; good communication is, therefore, essential for better understanding and addressing the needs of patients [18]. Nevertheless, the current curriculum for training PT students tends to emphasize professional knowledge and skills, without paying sufficient attention to cultivating their empathetic communication skills. Therefore, helping PT students develop empathetic communication skills before beginning their internship of clinical practice is a pressing issue. Communication skills can be trained, and one of the most fundamental types of training lies in the cultivation of empathy [10,19]. Moreover, the study of Tung and colleagues found scenario-based simulation training to be capable of enhancing the empathy of nursing professionals [20]. Such training enables nurses to vicariously experience the difficulties and senses of uncertainty and fear inflicting their patients, thereby making them more willing to think from patients’ perspectives and offer greater patience and support [20,21]. In addition to scenario-based simulation, reflective learning has also been found to help improve empathy in medical students [22,23]. By reflecting on their own experiences, students are more able to achieve a deeper understanding of the motivations behind their ideas, values, actions, and emotions, and proceed to develop new ideas, values, and thinking patterns; these, in turn, help facilitate self-growth, and guide behaviors in demonstrating empathy [22,23].

Drama in education, as a pedagogical design, is based on scenario simulation and reflective learning [24]. The structure and components of drama are incorporated into teaching, in order to help students achieve learning goals by engaging them in drama activities [25]. Other than training students to discover and raise questions, drama in education is able to encourage students to solve problems through self-reflection [26]. This study introduces the concepts of compound stimulus into drama in education, with the instructor offering guidance and clues to trigger students’ interest in understanding the person, and exploring the stories behind the person. Students are then invited to create their own stories for the person, and the stories are connected and organized into a complete narrative for students to practice role play. When playing out the person, students are able to experience the problems confronting the person, reach a deeper understanding of how the person must be feeling, imagine how they would try to solve the problems, and eventually achieve perspective taking [25]. With role play providing room for adding their own ideas and feelings, students are able to modify the personalities of the original person, and simulate how they react to the problems they are facing. Thus, role play brings about new stimuli, propelling students into constant thinking and reflection, as well as the pursuit for effective coping strategies or solutions, thereby improving their empathetic communication skills [24]. This study accordingly utilized the compound stimulus approach in the form of drama in education, in order to boost PT students’ empathetic communication skills and learn how to obtain accurate and precise information concerning the needs of their patients.

## 2. Materials and Methods

### 2.1. Study Design

**Figure 1:**
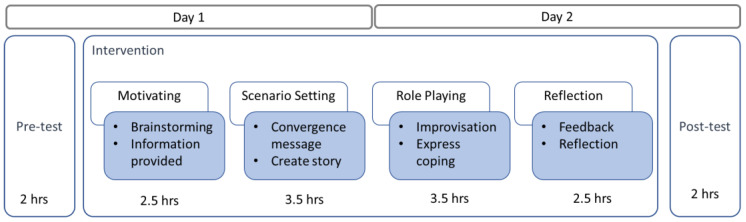
Research Process. Two-day workshop (16 hours) including pre-test, intervention, and post-test.

Based on a cross-sectional, one-group, pre- and post-test design, this study recruited junior (third-year) PT students to attend an intensive 2-day, 16-hour workshop, instead of a regular 16-week, 1-hour-per-week course. Collection of quantitative and qualitative data was conducted both before and after the workshop to help observe and examine the changes in students’ performance in terms of empathy and accuracy demonstrated through their clinical examination and history-taking skills. Students were asked to perform a self-assessment prior to joining the workshop, in order to prepare them for the subsequent post-workshop comparison. Please consult Figure 1 for illustration. 

### 2.2. Research Participants and Ethical Considerations

Research participants were 57 third-year students in the PT department of a medical university in Central Taiwan. Students needed to enroll in the course “Physical Therapy Clinical Internship” and sign an informed consent to become qualified participants. All research data were de-identified to safeguard participants’ right to personal privacy. For students choosing not to participate in the workshop, their right to learn was by no means affected in any manner.

### 2.3. Study Intervention

The workshop included three parts, a pre-test, a compound-stimulus-based intervention, and a post-test. Study participants were divided into 4 groups of 14-15 people per group for the two-day workshop, based on the concepts of compound stimulus in the form of drama in education. Four senior physical therapists (SPT) were invited to participate in the compound-stimulus-based intervention, due to their passion and rich work experience. A therapist led a group of students, and guided the students in performing the role play, group discussion, and feedback sharing. A compound stimulus teaching package was designed in the form of a suitcase containing a variety of artefacts as props and cues, such as a cane or notebook, which may be used by the case. The instructor could guide students to imagine the personality and daily life of the case from the props [17,25]. As indicated in Figure 1, the workshop unfolded in the following four stages:

(1) Motivating: The instructor sent out relevant clues and cues of the designed case to generate students’ interest in this case problem, such as walking problems after a stroke; this prompted students to start figuring out and establishing links between the who, why, where, when, and how of this case problem;
(2) Scenario Setting: The instructor guided students, group by group, to develop a story incorporating potential predicaments and problems that needed to be resolved;
(3) Role Playing: Each group of students created a story plot in an impromptu manner, connected potential predicaments and problems in a reasonable manner, and developed different but relevant clinical scenarios to begin the role play. Students took turns playing patient, therapist, or observers;
(4) Reflecting: Students shared with each other the scenarios, situations, or predicaments most notable to them, the personality traits and feelings of this case in the face of diseases, the problems this case would like to overcome, the outcomes they expected, and other related issues.

### 2.4. Instruments for Data Collection

Both before and after implementing the intervention in the form of a compound stimulus workshop, the study conducted an OSCE (Objective Structured Clinical Examination) to assess participants’ clinical examination and history-taking skills as applied to a standardized patient (SP) [27]. In order to ensure assessment objectivity by minimizing on-site interference, every OSCE assessment was recorded in its entirety and rated afterwards. According to the definition of empathetic communication, the students were to be able to listen to patients’ needs (for content and feelings), and to reflect their understanding to the patient using communication skills. Therefore, the study used the following measurements to test the students’ communication skills and empathy.

#### 2.4.1. Quantitative Data

Quantitative data of the study were collected based on the concepts of 360-degree evaluation and multi-dimensional assessment [2]. Feedback from multiple sources or multiple raters (i.e., the SP, the study participants, and the physical therapists) was assessed, and items assessed included the following: communication skills, demonstration of empathy, and professionalism (comprising accuracy of professional information, comprehensiveness of obtained information, and professional attitude). A total of five assessment instruments were adopted: Standard Patient Rating Scale (SPRS), Objective Structured Clinical Examination Scale (OSCES), Professional and Communication Self-Assessment Scale (PCSS), Patients’ Information (PI), and the Jefferson Scale of Empathy (JSE) (Table 1). 

##### Standard Patient Rating Scale (SPRS)

The SPRS is a self-developed scale incorporating three items rated on a 10-point scale (1 point = strongly disagree to 10 points = strongly agree). The three items relate to communication skills, demonstration of empathy, and professional attitude. The SP rated the three items based on their subjective perception right after the completion of a participant’s clinical examination. 

##### Objective Structured Clinical Examination Scale (OSCES)

The OSCES adopted by this study was a self-developed observational assessment tool using a 3-point Likert scale (0 = complete failure; 1 = partial achievement; and 2 = complete achievement). Based on the competencies that the Taiwan Physical Therapy Association expects of PT interns and clinicians, the scale incorporates 11 items on professionalism and 16 on the comprehensiveness of the information obtained (from the SP). The “professionalism” section is further divided into 5 items on communication (OSCES-C), 3 on empathy (OSCES-E), and 3 on attitude (OSCES-A). A higher score indicates a greater degree of achievement. The entire process of the OSCES was recorded, and the recorded assessment was graded by a senior physical therapist (SPT) in terms of OSCES-C, OSCES-E, and OSCES-A. The comprehensiveness of the information obtained (OSCES-I) was also assessed, in order to evaluate the overall clinical communication skills of the participants. According to a pilot study, the OSCES was considered a reliable and effective assessment instrument, with a content validity index of 0.8 and an inter-rater agreement of 85%. 

##### Professional and Communication Self-Assessment Scale (PCSS)

The PCCS was self-developed in this study for the participants to assess their own history-taking and clinical examination performance, with special attention directed to empathetic communication skills (9 items) and professional attitude (7 items). A 5-point scale is used, with 1 point indicating “strongly disagree” and 5 points indicating “strongly agree.” A higher score suggests a greater degree of self-confidence in empathetic communication and professional attitude. An item was added for participants to rate (by percentage) their self-confidence in the accuracy and comprehensiveness of the information they obtained. 

##### Patients’ Information (PI)

PI covered 20 questions concerning the background and situation of the SP. Participants asked the questions, and recorded the answers and information obtained from the SP. An SPT examined and rated the record, in order to evaluate the accuracy of the information obtained. A higher score indicated a greater degree of accuracy.

##### Jefferson Scale of Empathy (JSE)

As an instrument used to assess the empathy of healthcare providers, the JSE [28] has been translated into Chinese, and tested to report established reliability and validity [29]. The scale uses a 7-point Likert scale, with 1 point indicating “strongly disagree” and 7 points indicating “strongly agree.” The total score of the JSE falls in the range of 20~140 points; a higher score suggests a greater degree of empathy. The JSE has been extensively used by studies assessing the empathy of healthcare professionals in Taiwan [20,30]. 

#### 2.4.2. Reflection Diary

Participants were asked to keep a reflection diary through a written qualitative description of their thoughts about and responses to the workshop; there was no length restriction, and a feedback form was used for recording responses. 

### 2.5. Data Analysis

Descriptive statistics were used to analyze data about communication skills, demonstration of empathy, and professionalism (including the accuracy and comprehensiveness of the information obtained, and professional attitude). Changes in the score before and after participation in the workshop were examined using a paired *t*-test, while an independent sample *t*-test was used to examine the gender effect on all the variables. A *p*-value lower than 0.05 indicates statistical significance. The reflection diary, in the form of written data based on a qualitative description, was examined via simple qualitative content analysis (including reading, open coding, and categorizing), in order to develop a comprehensive understanding of participants’ feelings and feedback, as well as the influences of the workshop on participants.

## 3. Results

### 3.1. Basic Data/Variables of Participants

A total of 57 students participated in this study, including 28 males and 29 females, aged between 20.3 and 25.8 years (mean age 21.2 ± 1.2 years old). Please refer to Table 2 below for the pre- and post-test changes in variables.

### 3.2. Enhanced Professionalism

Professionalism incorporated professional attitude and the accuracy and comprehensiveness of the information obtained, and was assessed by an SPT (OSCES_A), the SP (SPRS_A), and the participants (students) themselves (PCSS_A). The results of the study indicate a significant improvement (*p* < 0.05) in all three aspects of assessment by the SPT, SP feedback, and the participants’ self-rated performance. A significant improvement was also observed in the accuracy (PI) and comprehensiveness of the information obtained (OSCES_I), suggesting that, after attending the workshop, participants were able to obtain more comprehensive and accurate information from their history taking and clinical examination of the SP. In terms of self-rated performance, participants demonstrated a significantly enhanced confidence in obtaining accurate patient information. Participants, in other words, found themselves more capable of understanding the SP after the workshop intervention.

### 3.3. Improved Communication Skills

Communication skills were assessed by an SPT (OSCES_C), feedback from the SP (SPRS_C), and the participants’ self-rated performance (PCSS_C), marking a significant improvement (*p* < 0.05) after the workshop intervention. The SP was more capable of understanding the questions and instructions from the participants, who, in turn, had learned to apply communication skills to their history taking and clinical examination based both on the opinion of the physical therapist and the self-perception of the participants. 

### 3.4. Higher Degree of Empathy

Empathy was also evaluated by an SPT (OSCES_C), feedback from the SP (SPRS_C), and the participants’ self-rated performance (JSE). Observing whether participants demonstrated empathy during clinical examination after they completed the workshop, the SPTs found that the SP felt more empathized with and was able to communicate with the participants with greater ease. Similarly, the participants found themselves expressing a higher degree of empathy while communicating with the SP. Both findings were statistically significant (*p* < 0.05), indicating the ability of the workshop intervention to boost empathy in the participants. 

### 3.5. Strong Inter-Rater Agreement 

In this study, the professional attitude, comprehensiveness and accuracy of the information obtained, and empathetic communication skills of the participants were assessed from multiple perspectives by an SPT, the SP, and the participants themselves. The results indicate that the SPT assessment of the participants’ professional attitude and demonstration of empathy and the SP assessment showed an agreement marked with a positive correlation (r = 0.40~0.52). The SPTs’ assessments of the comprehensiveness and accuracy of the information obtained by the participants reported a positive correlation with the SPTs’ assessments of participants’ professional attitude (r = 0.324), the VIP assessment of their communication skills (r = 0.264), and the participants’ self-rated performance (r = 0.449), thereby indicating strong SPT–participant inter-rater agreement (Table 3). 

### 3.6. Remarks on Gender Effect

According to the pre-test results, female participants scored significantly higher (*p* < 0.05) than male participants in terms of empathy shown to the SP. The significance, however, disappeared after the workshop intervention. As indicated by the post-test results, male participants were significantly more confident in the comprehensiveness and accuracy of the information they obtained, with an average score 10.5 points higher than that of their female counterparts. No significant gender difference was detected in the remaining variables (Table 4). 

### 3.7. Remarks on Participants’ Reflection Diary Feedback

Based on the feedback from the participants’ reflection diaries at the end of the workshop intervention, the teaching approach was innovative and beneficial in the following three aspects: improving learning effectiveness, enhancing communication and clinical examination skills, and increasing empathy toward patients. The following responses indicate improvement in learning effectiveness:

*“The ‘drama in education’ teaching approach adopted by the workshop is fairly innovative, showing me a brand new but effective way of better understanding the patient”; “with an approach different from that of regular courses, I learn more about how to proceed with clinical examination and modify my skills.”*

On the other hand, the following responses show how the workshop benefited the participants’ communication and clinical examination skills:

*“The workshop provides me with substantial help in improving my skills in clinical examination and communication with the patient”; “my clinical examination becomes better structured and more focused”; “I learn how to extract important information and imagine how the patient’s capacity is limited in daily life”; “I am able to obtain more comprehensive information from and better communicate with the patient”; “I develop a greater sense of direction that makes the questions I ask more logical.”*

For increasing empathy toward the patient, participants reported the following responses:

*“I’ve learned how to put myself in the patient’s place when performing clinical examination by observing his facial expressions to help me express the empathy expected of a physical therapist”; “According to the sharing and discussion of classmates in role playing, I’ve learned the communication skills needed to avoid using impolite or invasive words and body language so as to build trust in my interpersonal relationship with the patient”; “I’ve learned to adjust my posture and gesture during clinical examination in a way that shows my empathy toward the patient and his problems”; “I am able to use the patient’s language to communicate with him and pay more attention to the emotional responses of the patient and his family”; “I try to understand the potential problems that may be troubling the patient from his perspective and communicate with him in a way he is able to understand”; “I need to be empathetic to put myself into his shoes, steering clear of topics the patient may find offensive or too sensitive.”*

The above responses from the participants’ reflection diaries are in agreement with the results of the objective assessments on their professionalism, demonstration of empathy, and communication skills. 

## 4. Discussion

Compared to previous studies in the literature, this study is a rare case that applied the concepts of compound stimulus in the form of “drama in education” to the pre-internship education in the field of physical therapy. With its Objective Structured Clinical Examination employing a standardized patient, this study performed a multi-dimensional assessment to examine PT students’ performance in professionalism, empathy, and communication from the perspectives of a physical therapist, a standardized patient, and the participants themselves. The study results found that the compound-stimulus-based drama-in-education approach was capable of improving the empathetic communication skills of the participants, and strengthening their overall professionalism. An analysis of both the quantitative data and the participants’ reflection feedback suggests that the innovative teaching approach qualifies as a model for pre-internship PT education, as it encourages students to become more empathetic toward the patient, and enables them to adopt appropriate and effective ways to communicate with the patient, in order to obtain more accurate and comprehensive information. 

### 4.1. Promotion of Professional Competencies

The professional competencies that this study strives to promote include students’ professional attitude, and their ability to gather accurate and comprehensive information from the patient during clinical examination. As indicated by the findings of this study obtained from different raters, participants reported positive changes in their professional competencies after attending the workshop. From the perspective of SPTs, participants appeared to become more capable of acting like professional physical therapists, and more confident in understanding their patients. The findings are consistent with those of previous studies. Both scenario simulation [31,32,33] and communication skill courses [11] have been found to help trigger positive changes in students’ attitudes toward their patients, improve the understanding and knowledge of the patients, and boost confidence in communication. These positive changes may be caused by the role play in class and reflection after class [32]. Integrating the concepts of compound stimulus into drama in education provides students with a safe environment that allows them to play different roles, use different perspectives to examine the patients and situations they are facing, and examine the discrepancies between the theoretical knowledge they have learned and practical clinical applications, thereby enriching the depth of their learning [34,35]. By practicing reflection under the guidance of their instructor, students are able to compare theoretical knowledge to the experiences learned from role play, and develop new perspectives of interacting with their patients [36]. A learning experience such as this is more capable of generating greater motivation for students to achieve more profound levels of learning [33,37]. This study further shows that, as students practice deeper reflection, their improvement is not just a personal feeling, but is also in synch with the results of others’ observations. 

### 4.2. Improvement in Communication Skills and Demonstration of Empathy

In addition to improvement in communication skills, the findings of this study indicate that the participants reported a significant increase in their empathy score after completing the compound stimulus workshop in the form of drama in education. Similar findings can be observed in studies on communication courses [11,38], scenario simulation training [32], and role play activities [33]. In addition to improving their attitude toward patients and their professional competence, all the activities mentioned above enable health providers to demonstrate greater empathy toward their patients through improved provider–patient relationships (PPRs); the benefit can be further augmented if tailored training courses can be developed for medical professionals of different specialties [38]. 

According to Menezes and colleagues [39], communication courses, early clinical exposure, and reflective practice are all conducive to empathy training, and empathy can be promoted through role simulation and subsequent reflection and feedback sharing [32]. Early clinical exposure to a multiplicity of patents provides students with opportunities to interact with patients coming from different backgrounds and experiencing different health problems. This, in turn, enables students to recognize the need to understand the different feelings of patients, as well as the different ways of expressing those feelings, and the importance of responding to those feelings with empathy [23]. As pre-internship students have not yet had the chance to directly interact with patients, this study’s compound-stimulus-based drama-in-education workshop offers them an environment for simulating clinical examination and experiencing direct interaction with patients. With SPT guidance, role play, simulated clinical examination, and reflective feedback sharing, students learn to comprehend the problems confronting their patients, empathize with the emotional changes or even mood swings of the patients, and recognize how important their words and actions can be to the patients. All of these factors help students reach a better and deeper understanding of the influences of language and emotion on PPRs, which serves as the basis to figure out what they need to improve in their communicative behavior, and how to better empathize with and respond to their patients [23,39].

Learning from positive role models also helps cultivate empathy [23]. This study’s compound-stimulus-based “drama-in-education” workshop was conducted with the assistance of four professional physical therapists. Three of the four physical therapists were alumni of the physical therapy program who volunteered to assist in the workshop during off-duty hours. The importance and value of dedication were demonstrated to the participants via the professionalism and passion of their alumni. Moreover, in the face of the alumni’s vivid interpretation of a patient’s situations and feelings, the participants learned that they were witnessing something that could not have been achieved without an in-depth understanding of, and profound empathy with, the patient. Therefore, finding positive role models is an effective way of fostering empathy [23].

Essential components of the workshop included the interactive theater, role play, group discussion, and feedback sharing, all of which helped not only keep classroom distraction at bay, but also prompted students into raising questions for active participation. Vigorous Q&A interaction led to valuable experience, thus achieving the goal of active learning. A total of 95% of the participants found the workshop to be an innovative teaching approach that was more effective than the traditional clinical practicum in helping them improve empathetic communication skills. 

### 4.3. Effects of Gender on Learning Outcome

According to the study’s results, prior to attending the workshop, female participants demonstrated a higher level of empathy than male participants, a finding consistent with the one reported in a previous study that found female PT majors to be more empathetic [12]. In this study, after attending the workshop, both male and female participants showed an improvement so significant that the previous obvious discrepancy in empathy between the two groups was no longer detected, suggesting that male students were able to cultivate empathy through learning, and demonstrated a degree of improvement exceeding that of their female counterparts. This finding, however, is inconsistent with the result of an earlier study. While training helped students of both genders enhance the level of empathy, female students emerged superior in terms of empathy, both before and after training in the study by Dorough and colleagues [38]. When it comes to the comprehensiveness and accuracy of the information obtained from patients during clinical examination, a similar increase in post-workshop self-confidence was observed in participants of both genders in this study; however, male participants reported a significantly higher score for self-confidence than female participants after the intervention. This result is in agreement with a result reported in the literature. As indicated by the study by Blanch and colleagues [40], though their performance emulated that of their male counterparts, female students appeared to be less confident in themselves. Therefore, helping promote clinical confidence in female students is an important issue worth studying.

### 4.4. Implications and Applications of the Study

In this study, the demonstration of empathy [41,42], communication skills, and professional competence of PT students were assessed simultaneously from different perspectives of different raters (i.e., SPTs, SP, and participants themselves). This application of 360-degree evaluation and multi-dimension assessment is rare in the literature. Furthermore, this study engaged a standardized patient for participants to simulate clinical examination in an objective and structured manner. Each examination was recorded in its entirety, in order for an SPT to watch the recorded video and rate the participant’s performance at length and leisure, thus helping to minimize on-site interferences that could affect assessment accuracy and objectivity. This approach may be considered for application in future assessments. Moreover, experienced clinical physical therapists were sent to assist in the development of the assessment instruments, namely, the OSCES and PCSS, helping to convert the essential clinical examination skills students were expected to learn into objective, concrete criteria for quantitative assessment. Both scales provide participants with valuable tools for assessing whether they reached the expected competencies in their future clinical internship.

### 4.5. Research Limitations

As a result of the COVID-19 pandemic, all participants were unable to complete the clinical examination in the latter part of the study period. SPTs were accordingly unable to assess the OSCE performance of these participants, who in turn could not conduct the PCSS self-assessment for future reference. The other limitation was that there were multiple components to the teaching package, making it difficult to determine which components had more or less influence on the intervention.

## 5. Conclusions

This study developed and implemented an innovative teaching model, using drama-in-education simulation, role play, and reflective practice, to help participants experience interactions with patients during clinical examination, improve their communication skills, and foster their empathy. The teaching model can be expected to provide insights in the development of training courses for pre-internship PT students. The OSCES and PCSS developed in this study can also be expected to provide both instructors and students with valuable tools for assessing clinical examination skills in future internships.

## Figures and Tables

**Table 1:** Overview of quantitative assessment instruments adopted by the study.

Resources	Instrument (No. of Items)	Communication (No. of Items)	Empathy (No. of Items)	Professionalism (No. of Items)		
				Attitude	Information: Comprehensiveness	Information: Accuracy
Feedback from SP	SPRS (3)	SPRS_C (1)	SPRS_E (1)	SPRS_A (1)		
Professional Assessment	OSCES (28) PI (20)	OSCES_C (5)	OSCES_E (3)	OSCES_A (3)	OSCES_I (16)	P I(20)
Participant Self-Assessment	PCSS (14) JSE (20)	PCSS_C (6)	JSE (20)	PCSS_A (7)	PCSS_I (1)	

**Table 2:** Pre- and post-test changes in research variables.

Variables	Pre-Test	Post-Test	Changes	t-Value	*p*-Value
SPRS_Sum	18.2 (3.4)	24.8 (2.9)	6.5 (3.1)	−15.98	<0.001
SPRS_A	5.5 (1.5)	8.5 (1.1)	2.9 (1.3)	−16.57	<0.001
SPRS_C	7.2 (1.0)	8.2 (1.0)	1.0 (1.1)	−7.20	<0.001
SPRS_E	5.5 (1.8)	8.1 (1.3)	2.6 (1.8)	−10.63	<0.001
OSCES_Sum	10.9 (2.7)	18.0 (3.0)	7.1 (2.4)	−22.48	<0.001
OSCES_A	2.9 (1.3)	4.7 (1.6)	1.8 (1.4)	−9.81	<0.001
OSCES_C	3.6 (0.9)	5.4 (0.9)	1.8 (1.1)	−12.48	<0.001
OSCES_E	4.4 (1.2)	7.8 (1.5)	3.5 (1.5)	−17.90	<0.001
OSCES_I	10.9 (4.9)	17.8 (3.8)	6.9 (5.2)	−10.02	<0.001
PI	16.5 (5.3)	23.7 (4.6)	7.1 (6.2)	−8.74	<0.001
PCSS_Sum	45.7 (6.1)	54.7 (5.0)	9.0 (5.8)	−11.61	<0.001
PCSS_A	24.7 (3.6)	29.6 (2.8)	4.9 (3.4)	−10.84	<0.001
PCSS_C	21.0 (2.8)	25.1 (2.6)	4.0 (3.0)	−10.18	<0.001
PCSS_I	46.7 (16.0)	71.0 (13.8)	24.3 (16.7)	−11.00	<0.001
JSE	110.7 (11.8)	114.1 (12.3)	3.4 (8.1)	−3.16	0.003

SPRS: Standard Patient Rating Scale; OSCES: Objective Structured Clinical Examination Scale; C: Communication; E: Empathy; A: Attitude; I: Information; PCSS: Professional and Communication Self-Assessment Scale; PI: Patients’ Information; JSE: Jefferson Scale of Empathy.

**Table 3:** Correlations between SP and SPT assessment results.

SPT/Patient/ Participant	Post_OSCES_A	Post_OSCES_E	Post_OSCES_C	Post_ OSCES_I	Post_PI
Post_ SPRS_A	0.401 **	0.516 **	0.055	0.269 *	0.324 *
Post_ SPRS_C	0.238	0.178	0.071	0.357 **	0.264 *
Post_ SPRS_E	0.472 **	0.393 **	0.067	0.179	0.207
Post_PCSS_A	0.01	0.124	−0.004	0.093	0.250
Post_PCSS_C	0.048	0.030	−0.123	−0.124	0.296 *
Post_PCSS_I	−0.074	0.141	−0.022	0.075	0.449 **
Post_JSE	0.185	0.196	0.223	0.210	0.189

* *p* < 0.05; ** *p* < 0.01.

**Table 4:** Gender difference: pre-test vs. post-test comparison.

	Pre-Test			Post-Test		
	Male	Female	*t*-Value	Male	Female	*t*-Value
SPRS_Sum	17.4 (3.6)	19.1 (3.1)	1.93	24.5 (2.9)	25.0 (2.8)	0.66
SPRS_C	7.1 (1.0)	7.3 (0.9)	0.80	8.1 (1.0)	8.3 (0.9)	1.16
SPRS_E	5.0 (2.0)	6.0 (1.5)	2.15 ^*^	8.0 (1.4)	8.2 (1.2)	0.51
SPRS_A	5.3 (1.6)	5.8 (1.4)	1.27	8.4 (1.1)	8.5 (1.2)	0.19
OSCES_Sum	10.0 (2.7)	10.9 (2.7)	0.06	18.14 (3.3)	17.76 (3.0)	−0.48
OSCES_C	3.5 (1.0)	3.6 (0.9)	0.21	5.4 (0.9)	5.4 (0.9)	−0.06
OSCES_E	4.4 (1.2)	4.4 (1.2)	0.17	7.9 (1.5)	7.9 (1.5)	0.01
OSCES_A	3.0 (1.5)	2.9 (1.2)	−0.18	4.9 (1.4)	4.5 (1.8)	−0.90
OSCES_I	9.8 (5.2)	12.0 (4.5)	1.67	17.3 (3.8)	18.3 (3.8)	1.09
PI	15.8 (2.9)	17.2 (4.7)	1.07	23.2 (5.4)	24.1 (3.7)	0.70
PCSS_Sum	45.4 (6.5)	46.0 (5.8)	0.42	55.4 (4.8)	53.9 (5.2)	−1.12
PCSS_C	20.9 (3.1)	21.2 (2.6)	0.42	25.3 (2.3)	24.8 (2.8)	−0.78
PCSS_A	24.5 (3.8)	24.9 (3.4)	0.38	30.1 (2.7)	29.1 (2.8)	−1.33
PCSS_I	48.4 (16.6)	45.0 (15.6)	−0.80	76.3 (10.0)	65.8 (15.0)	−3.01 *
JSE	107.7 (13.6)	113.6 (9.1)	1.94	112.6 (15.0)	115.5 (8.9)	0.90

* *p* < 0.05.

## Data Availability

These study data are identified participant data. The data that support the findings of this study are available, beginning 12 months and ending 36 months following the article publication from the corresponding author, Y.-H.C, upon reasonable request at yhchu@mail.cmu.edu.tw.

## References

[1] Tsai M.W., Jeng S.F., Wang S.F. (2022). Establishment of the Six-Year Doctor of Physical Therapy Education in Taiwan. Formosan J. Med..

[2] Wu Y.T. (2013). Teaching and Learning in Physical Therapy.

[3] Physiotherapy W. Physiotherapist Education Framework. https://world.physio/what-we-do/education.

[4] Liu W.Y., Lien H.Y., Li Y.C., Chen C.N., Chen C.Y., Chen Y.C., Cheng C.H., Lin Y.H. (2019). Core Values of Physical Therapy Professionalism: The Viewpoints of Clinical Physical Therapists in Taiwan. FJPT.

[5] Hsu S.S., Wu Y.T., Chien M.Y., Ling W., Hu M.H. (2007). Entry Level Education and Clinical Education for Physical Therapy in Taiwan. J. Med. Educ..

[6] Tsai M.S., Lai C.S., Huang W.Y., Wu Y.T. (2009). Current Status and Critical Reflection of Physical Therapy Education Program for Junior Colleges in Taiwan. FJPT.

[7] Cobley P., Donsbach W. (2008). Communication: Definitions and Concepts. The International Encyclopedia of Communication.

[8] Huang L.L. (2018). From Sympathy to Empathy—Is It Possible to Have a Good Physician-Patient Communication without “Fusion of Horizons”?. Philos. Cult..

[9] Gao M.J. (2015). Doctor-patient communication should strengthen empathy communication skills courses and training. Taiwan Med. J..

[10] Drossman D.A., Chang L., Deutsch J.K., Ford A.C., Halpert A., Kroenke K., Nurko S., Ruddy J., Snyder J., Sperber A. (2021). A Review of the Evidence and Recommendations on Communication Skills and the Patient-Provider Relationship: A Rome Foundation Working Team Report. Gastroenterology.

[11] Boissy A., Windover A.K., Bokar D., Karafa M., Neuendorf K., Frankel R.M., Merlino J., Rothberg M.B. (2016). Communication Skills Training for Physicians Improves Patient Satisfaction. J. Gen. Intern. Med..

[12] Holmes M.B., Driscoll L., Murphy E., Starr J.A. (2019). A Cross-Sectional Study of Empathy Among Students at Two Doctor of Physical Therapy Programs in Boston. J. Allied Health.

[13] Rodriguez-Nogueira O., Leiros-Rodriguez R., Pinto-Carral A., Alvarez-Alvarez M.J., Morera-Balaguer J., Moreno-Poyato A.R. (2022). The association between empathy and the physiotherapy-patient therapeutic alliance: A cross-sectional study. Musculoskelet. Sci. Pract..

[14] Magalhaes E., Salgueira A.P., Costa P., Costa M.J. (2011). Empathy in senior year and first year medical students: A cross-sectional study. BMC Med. Educ..

[15] Khademalhosseini M., Khademalhosseini Z., Mahmoodian F. (2014). Comparison of empathy score among medical students in both basic and clinical levels. J. Adv. Med. Educ. Prof..

[16] Chen D.C., Kirshenbaum D.S., Yan J., Kirshenbaum E., Aseltine R.H. (2012). Characterizing changes in student empathy throughout medical school. Med. Teach..

[17] Somers J. (2008). Interactive theatre: Drama as social intervention. Music. Arts Action.

[18] Association A.P.T. What Physical Therapists Do. https://www.apta.org/your-career/careers-in-physical-therapy/becoming-a-pt.

[19] Srivastava U., Price A., Chu L.F. (2021). Effects of a 2-Week Remote Learning Program on Empathy and Clinical and Communication Skills in Premedical Students: Mixed Methods Evaluation Study. JMIR Med. Educ..

[20] Tung L.C., Chang L.C. (2017). Effect of Scenario-Based Simulation Training on Nurse Empathy. J-TSSH.

[21] Del Vecchio A., Moschella P.C., Lanham J.G., Zavertnik J.E. (2022). Acting to teach communication skills to nurses. Clin. Teach..

[22] Leung K.K. (2015). The Roles of Reflection in Medical Education. Taiwan J. Fam. Med..

[23] Seeberger A., Lonn A., Hult H., Weurlander M., Wernerson A. (2020). Can empathy be preserved in medical education?. Int. J. Med. Educ..

[24] Cheng Y.H., Wu Y.J. (2015). “Play” Together: Drama Performance in Character Education: A Case Study in Taiwan. J. Chin. Creat..

[25] Chang H.H. (2014). Drama in Educaiton for Transdisciplinary Domain: The Curriculum Design and Praxis.

[26] Pan S.T. (2020). Teaching Innovation of Lecturers in colleges: Practice and Reflection Based on Action Research. J. Taiwan Educ. Stud..

[27] Tsai S.L., Chen C.H., Fang J.T., Tsai J.J., Chang S.C. (2008). A Guideline for the Implementation of Objective Structured Clinical Examination. J. Med. Educ..

[28] Hojat M., Gonnella J.S., Nasca T.J., Mangione S., Vergare M., Magee M. (2002). Physician empathy: Definition, components, measurement, and relationship to gender and specialty. Am. J. Psychiatry.

[29] Cheng J.F., Lai Y.M., Livneh H., Tsai T.Y. (2011). Establishing Reliability and Validity of the Chinese Version of the Jefferson Scale of Empathy. J. Nurs..

[30] Kuo J.C., Cheng J.F., Chen Y.L., Livneh H., Tsai T.Y. (2012). An exploration of empathy and correlates among Taiwanese nurses. Jpn. J. Nurs. Sci..

[31] Cheng W.L.S., Ma P.K., Lam Y.Y., Ng K.C., Ling T.K., Yau W.H., Chui Y.W., Tsui H.M., Li P.P. (2020). Effects of Senior Simulation Suit Programme on nursing students’ attitudes towards older adults: A randomized controlled trial. Nurs. Educ. Today.

[32] Lin I.H., Wang C.Y., Lin Y.N., Chen H.C., Lin L.F. (2022). Simulation-based holistic education in physiotherapy interns to increase empathy toward older adults and individuals with disabilities. BMC Geriatr..

[33] Cortes-Rodriguez A.E., Roman P., Lopez-Rodriguez M.M., Fernandez-Medina I.M., Fernandez-Sola C., Hernandez-Padilla J.M. (2022). Role-Play versus Standardised Patient Simulation for Teaching Interprofessional Communication in Care of the Elderly for Nursing Students. Healthcare.

[34] Arveklev S.H., Wigert H., Berg L., Burton B., Lepp M. (2015). The use and application of drama in nursing education--an integrative review of the literature. Nurse Educ. Today.

[35] Middlewick Y., Kettle T.J., Wilson J.J. (2012). Curtains up! Using forum theatre to rehearse the art of communication in healthcare education. Nurse Educ. Pract..

[36] Mann K., Gordon J., MacLeod A. (2009). Reflection and reflective practice in health professions education: A systematic review. Adv. Health Sci. Educ. Theory Pract..

[37] Sobral D.T. (2000). An appraisal of medical students’ reflection-in-learning. Med. Educ..

[38] Dorough R.J.M., Adamuti-Trache M., Siropaides C.H. (2021). Association of Medical Student Characteristics and Empathy After a Communication Workshop. J. Patient Exp..

[39] Menezes P., Guraya S.Y., Guraya S.S. (2021). A Systematic Review of Educational Interventions and Their Impact on Empathy and Compassion of Undergraduate Medical Students. Front. Med..

[40] Blanch D.C., Hall J.A., Roter D.L., Frankel R.M. (2008). Medical student gender and issues of confidence. Patient Educ. Couns..

[41] Singer T., Klimecki O.M. (2014). Empathy and compassion. Curr. Biol..

[42] Klimecki O., Singer T. (2012). Empathic distress fatigue rather than compassion fatigue? Integrating findings from empathy research in psychology and social neuroscience. Pathol. Altruism.

